# Editorial: Neurorobotics explores the human senses

**DOI:** 10.3389/fnbot.2023.1214871

**Published:** 2023-05-22

**Authors:** Mehdi Khamassi, Marco Mirolli, Christian Wallraven

**Affiliations:** ^1^Institute of Intelligent Systems and Robotics, Sorbonne Université, CNRS, Paris, France; ^2^Institute of Cognitive Sciences and Technologies, National Research Council, Rome, Italy; ^3^Department of Brain and Cognitive Engineering, Korea University, Seoul, Republic of Korea; ^4^Department of Artificial Intelligence, Korea University, Seoul, Republic of Korea

**Keywords:** neurorobotics, human-robot interaction, central pattern generator, developmental robotics, machine learning, locomotion, autism spectrum disorder, visual and auditory perception

## 1. Introduction

The present Research Topic of Frontiers in Neurorobotics, entitled “*Neurorobotics explores the human senses*,” presents a variety of research studies at the crossroads between Neuroscience, Developmental Psychology, Artificial Intelligence (AI), and Robotics. The main common point of these studies is the need to better understand how humans (and animals in general) perceive their surrounding world and use this knowledge for Robotics. For human-robot interaction applications, this requires understanding how humans perceive and react to different robots and their behaviors. For applications to autonomous robots, this implies taking inspiration from the way humans perceive and react to different types of stimuli.

Research in AI and Robotics has always drawn at least some degree of inspiration from biology and human cognition. Classical examples illustrating this inspiration include the design and testing of humanoid robots, whose body is inspired by human morphology. Another striking example is the research on deep neural networks, which are loosely inspired by biological neurons in the brain, whose connections are modified through learning as the synapses between real neuron. Furthermore, some of the current research in AI takes inspiration from the human brain's cognitive architecture (as it is currently understood) (LeCun, 2022), and from the mechanisms of this architecture that contribute to the high level of behavioral flexibility and the fast learning abilities of humans (Hassabis et al., 2017; Alexandre et al., 2020).

Indeed, the whole field of Neurorobotics aims at mimicking some of the physical, behavioral and even neural properties of animals' body, brain activity, and behavior (Floreano et al., 2014). One of the goals of this field is to develop a new generation of robots that can interact with their environment in a more adaptive and flexible way by drawing inspiration from the functioning and organization of the nervous system. Another important goal is to contribute to a better understanding of how the human nervous system works by testing computational neuroscience models in real robots.

Interestingly, around the year of birth of the journal *Frontiers in Neurorobotics*, that is 2007, a series of papers advocated the dual contribution of Neurorobotics research to both Neuroscience and Robotics (Pfeifer et al., 2007; Arbib et al., 2008; Meyer and Guillo, 2008).

One advantage is: by incorporating knowledge from neuroscience into the design and control of robots, researchers can develop more sophisticated and efficient control algorithms that allow robots to perform tasks that are currently beyond their reach. Nevertheless, a converse objective of Neurorobotics research which is often underappreciated is the contribution to modeling, to simulating, and in the end to better understanding human behavior and cognition.

There are several ways in which Neurorobotics research can make specific contributions to Neuroscience and Psychology. One is about the role of embodiment. Testing computational models on real robots often leads to new observations and new understanding of the dynamics of sensorimotor coupling between the robot and its environment. This approach can allow us to go beyond perfectly controlled computer simulations by sometimes showing solutions that do not work in the real world, by uncovering new unanticipated problems, or by revealing previously overlooked properties of the body-environment coupling: by doing so, we can gain a deeper understanding of how systems behave under more realistic conditions, leading to more robust and reliable solutions. One of the most beautiful examples is the research on passive dynamic walkers, where a physical body constituted of metal legs and knees can produce a seemingly natural and smooth walking on an inclined plan even without being controlled by a computer (Collins et al., 2001). This strikingly illustrates that the walking problem should not be fully solved through neural computation, and that part of the solution rather lies in the physical coupling of the body with the environment.

Another way in which Neurorobotics research can contribute to Neuroscience and Psychology is by raising novel hypotheses. Often, computational models first designed and tested in simulations, and later transferred to real robots, do not work in the first place. This leads to modifications or additions of mechanisms to make the system work. Once successful modifications are found, the modified system can constitute a novel hypothesis for neuroscience or psychology, which can later be tested in these fields. For instance, previous work controlling a humanoid robot by a neuro-inspired prefrontal cortex model to learn from positive and negative feedback led to the question of which salient signals in the environment should be interpreted by the robot as feedback (Khamassi et al., 2011). In order not to give the solution a priori to the robot, the authors tested a dopamine-based reinforcement mechanism by which simulated dopamine neurons would respond to any salient event during the task, but would lead to reinforcement only when these responses are contingent on the arrival of a neural signal representing a motor efference copy, which means that the robot had actually performed an action and needed an evaluation for that action. Not only did this modified model produce efficient behavior for the tasks at hand. At the neural level, it constituted a novel hypothesis about dopamine neurons' activity which could later be tested through new neuroscience experiments.

The last possible contribution of Neurorobotics research which is particularly well illustrated by the articles in this Research Topic is the production of new research questions. For example, making a robot interact with humans leads to the question of how humans react to the robot, to its voice, to its actions, or simply whether humans succeed in recognizing the emotion mimicked by the robot. Combining a learning model and a perception model in a real robot, which is required to make it learn from real stimuli, leads to the question of how perception and learning shall interact. Finally, controlling a robot by a neural network to make it rhythmically walk leads to the question whether the same traditional action selection mechanisms used to select discrete non-periodic actions can be reused for the selection of periodic actions.

One of the important lessons learned from years of Neurorobotics research is that one of the best ways to answer the questions raised by the field is to directly address them: designing a specific protocol to answer the question, making a series of experiments, for instance with various human participants interacting with a robot, and measuring how human participants behave, react, or simply sense the various elements of the situation. This is a direction of research where roboticists have developed stronger collaborations with neuroscientists and psychologists, in order to learn how to design rigorous protocols and rigorous ways to measure and assess human behaviors. This is well-illustrated by several of the articles in this Research Topic.

## 2. Contributions to the Research Topic

In the paper “The Human Takes It All: Humanlike Synthesized Voices Are Perceived as Less Eerie and More Likable. Evidence From a Subjective Ratings Study,” Kühne et al. investigate the subjective evaluation of three different types of voices: two computer-generated text-to-speech systems vs. a control, human voice. The motivation for this study is not only to look for differences between synthesized and natural voices, but also to look for potential evidence of the occurrence of a so-called “Uncanny Valley” effect in these ratings. In the visual domain, this effect is well-documented, referring to a sudden increase in subjectively-perceived “eeriness” of the appearance of a humanoid entity as it starts to become more human-like. This effect has been made responsible, for example, to the reception and following re-design of characters in “Alina” or “Sonic The Hedgehog.” Interestingly, the authors here found no clear evidence for such an effect in the auditory domain within the range of tested voice stimuli—as human-likeness ratings increased, the corresponding eeriness ratings decreased with no sudden reverse effect. The study in addition establishes the human, control voice as a clear “winner” in the subjective evaluation, indicating that there is still a gap for these systems in terms of realism. Kühne et al. also include several interesting exploratory and qualitative analyses of the ratings and meta-information that provide a richer context for potential effects of external information on auditory processing and evaluation.

The paper “Do different robot appearances change emotion recognition in children with ASD?” by Pinto-Bernal et al., reports on the final stage of a participatory design process through which the authors have developed a robot (named “Castor”) that can be used as a therapeutic aid with children with autism. The final stage of the participatory design, which took place in a Colombian clinic, involved two steps. First, 50 different sketches of possible robot appearance divided in five categories (cartoon persons, traditional robots, futuristic characters, animal-like appearance, and fantasy characters) were evaluated by volunteers including caregivers and parents, but not the children. From the nine best sketches resulting from this first evaluation, three were selected by a second selection process involving nineteen children with autism spectrum disorder (ASD): one robot-like, one fantastic-like, and one human-like. Finally, the three resulting appearances were tested in an experiment in which 21 children with ASD were given an emotion recognition and imitation task. In particular, children had to recognize and imitate four different emotions (happiness, fear, sadness, and anger) expressed either by a cartoon in a card (control condition) or by one of the three selected robots. Very good performance was found with happiness and sadness, while fear and anger were recognized less well. While the results show no statistically significant difference in performance for the three appearances, the robot-like appearance was the one receiving more attention from the children, and, compared to the control condition, the presence of a robot, in general, increased the amount of attention demonstrated by the children.

The paper “A Database for Learning Numbers by Visual Finger Recognition in Developmental Neuro-Robotics” by Davies et al. deals with the topic of endowing an artificial system with the visual skills necessary for counting from images of human or robot hands. The context and motivation of the article are rooted in Developmental (Neuro)Robotics, in which emphasis is placed on naturalistic training and application scenarios with a particular focus on, for example, the embodiment of a robotic system. Signaling numbers by means of hand gestures is a deeply-rooted and important strategy both for teaching and communication in humans and hence this work has an immediate connection to the target of the special collection. To this end, Davies et al. create a dataset of hand postures covering the digits “1” to “5” being signed by both robot and human hands in various postures. The authors test various artificial neural network systems on their data, taking care to report a full range of performance measures including confusions and computational cost, which allow for a better evaluation of the efficacy and effectiveness of the different approaches. Overall, the purely visual analysis of these images results in good recognition performance—in addition, the typical errors of the algorithms are shown to also represent challenging cases for human interpretation. The article has made the dataset and analysis tools openly available, adding a valuable resource to the field for further experimentation.

Finally, the paper by Suzuki et al. (“Sprawling Quadruped Robot Driven by Decentralized Control With Cross-Coupled Sensory Feedback Between Legs and Trunk”) highlights the importance of sensory feedback in locomotion. Building on previous work where the authors had demonstrated sprawling behavior in a simulated quadruped robot, they test here their approach in a real, newly developed robot with nine actuated degrees of freedom (one in the trunk and two in each of the four legs). Taking inspiration from what is known about sprawling locomotion in vertebrate animals like salamanders, the controller consists in a series of oscillators representing central pattern generators that, in contrast with most previous models involving open-loop control, are sensory-coupled through bidirectional feedback between the legs and the trunk. The results show that a controller endowed with this cross-coupled sensory feedback is faster and more energy-efficient than one without it. Furthermore, the authors demonstrate that their system is also robust to morphological changes (showing stable locomotion with longer legs without changes in control parameters) and even to leg failure (i.e., when a leg is kept fixed in a given position). These results underline the pivotal role played by sensory feedback in the coordination of the body and the limbs.

## 3. Conclusions

From our point of view, these four papers well illustrate the common interest in neuroscience and psychology that the neurorobotics community shares even when the applications are different. In this respect, it is striking that this common interest, and the sharing of some of the methods to study human (and animal) behavior, have recently brought the neurorobotics community and the human-robot interaction (HRI) community closer to each other. Researches in HRI (Delaherche et al., 2015) and Human-Computer Interaction (HCI) (Bailly et al., 2022) seem to pay a growing attention to computational neuroscience models. Conversely, researchers in neurorobotics consider more and more often HRI scenarios as interesting testbeds of theirs models (Dromnelle et al., 2022). We think that human-robot interaction and animal-robot interaction studies constitute a promising paradigm to further our understanding of human and animal senses and improve robot senses accordingly.

## Author contributions

All authors listed have made a substantial, direct, and intellectual contribution to the work and approved it for publication.
